# Downward trend in the prevalence of *Helicobacter pylori* infections and corresponding frequent upper gastrointestinal diseases profile changes in Southeastern China between 2003 and 2012

**DOI:** 10.1186/s40064-016-3185-2

**Published:** 2016-09-19

**Authors:** Jian-Xia Jiang, Qing Liu, Xin-Yi Mao, Hai-Han Zhang, Guo-Xin Zhang, Shun-Fu Xu

**Affiliations:** 1 Department of Gastroenterology, The First Affiliated Hospital of Nanjing Medical University, No. 300, Guangzhou Road, Nanjing, 210029 China; 2 Department of Gastroenterology, Sir Run Run Hospital Nanjing Medical University, No. 109, Longmian Road, Nanjing, 211199 China; 3 Department of Gastroenterology, The Second People’s Hospital of Wuhu, Wuhu, China

**Keywords:** Epidemiology, *Helicobacter pylori* infection, Gastrointestinal diseases

## Abstract

This present study aims to determine trends in the prevalence of *H. pylori* infections in Southeastern China between 2003 and 2012, and investigate corresponding changes in the prevalence of upper gastrointestinal diseases. This retrospective study screened 196,442 patients with a mean age of 47.49 ± 14.47 years (age range 5–100 years) in Southeastern China, and a total of 134,812 cases of an endoscopy-referral patient population with digestive symptoms between 2003 and 2012 were enrolled. Based on esophago-gastro-duodenoscopy and pathology, patients diagnosed with chronic gastritis, peptic ulcer, gastric cancer or reflux esophagitis were included in this study. Basic demographic and clinical characteristics such as *H. pylori* infection status and endoscopic findings were collected and analyzed. Among the 134,812 subjects, mean prevalence of *H. pylori* infection was 31.97 %; which demonstrated a linear downward trend from 42.40 to 23.82 % (*P* < 0.001) at an annual rate of 2 % from 2003 to 2012. Similarly, the prevalence of duodenal and gastric ulcer rapidly decreased from 12.65 to 6.57 % and from 7.51 to 3.78 %, respectively; while the prevalence of gastric cancer (from 3.76 to 2.34 %) did not significantly change in the same time period. In contrast, the prevalence of reflux esophagitis increased from 6.19 to 12.80 %. The progressively decreasing prevalence of *H. pylori* infections from 2003 to 2012 in Southeastern China appears to be linked with the decline of related upper gastrointestinal diseases and increase of some gastrointestinal motility diseases.

## Background

Since the first description of *H. pylori* by Warren and Marshall (1983), bacterium has been thought to be one of the most common human infections worldwide. It was believed that it affected approximately half of the world’s population with geographic prevalence variations (Cover and Blaser 2009). Initially, the prevalence of *H. pylori* infections was described as nearly 70–90 % in developing countries, and approximately 25–50 % in developed countries (Taylor and Parsonnet 1995). A retrospective analysis of endoscopic data performed in the US revealed a 65.8 % *H. pylori* infection rate from 1993 to 1998 (McJunkin et al. 2011). Age-adjusted prevalence of *H. pylori* infections reached as high as 70.5 % as revealed by serum *H. pylori* antibody tests in Japan in 1988 (Nakajima et al. 2010). One study from South Korea revealed that the *H. pylori* infection rate determined by rapid urease test (RUT) was 50.0 % in 1997 (Lee et al. 2007). However, a meta-analysis in China in 1991 revealed that the prevalence of *H. pylori* infection was 49.4 % among 15- to 22-year-old healthy volunteers and 64.5 % in 13- to 88-year-old symptomatic endoscopically treated patients (Li et al. 1991). Another study reported that the infection rate of subjects who underwent routine health examinations in Guangzhou, China in 1992 was up to 52.4 % as shown by enzyme-linked immunoabsorbent assays (ELISA) (Mitchell et al. 1992).

Numerous studies have made it clear that *H. pylori*-positive and *H. pylori*-negative subjects have substantial differences in gastric physiology, as well as in tissue and immune responses (Blaser 2005). *H. pylori* is widely considered to be the most important etiologic factor in peptic ulcer disease (Tytgat et al. 1993), and is linked etiologically to non-cardia gastric cancer (Helicobacter and Cancer Collaborative Group 2001) and gastric mucosa-associated lymphoid tissue lymphoma (Stolte et al. 2002). Nowadays, the diagnosis and therapy of *H. pylori* infections has become a standard practice in the clinic. According to several international guidelines in the last two or three decades, triple and quadruple, sequential, or concomitant therapy regimens are recommended for *H. pylori* eradication therapy, which led to eradication rates of 71–89 % (Hossenini et al. 2014).

In recent years, the prevalence of *H. pylori* infections has progressively declined worldwide. One cross-sectional study in the US that compared endoscopic *H. pylori* prevalence between 1993–1998 and 2004–2009 revealed that infection rates substantially decreased (McJunkin et al. 2011). Although many studies have focused on the changes of *H. pylori* infection over time, long-term trends combined with changes in the prevalence profile of frequent upper gastrointestinal diseases (UGIDs) related to *H. pylori* infections have been rarely studied (McJunkin et al. 2011; Lee et al. 2007; Li et al. 1991). Therefore, this present large-scale, retrospective study was initiated to investigate trend changes in the prevalence of *H. pylori* infections, as well as corresponding alterations of the prevalence of UGIDs frequently associated with *H. pylori* infection in Southeastern China.

## Results

### Baseline characteristics and changes in the prevalence of *H. pylori* infection in an endoscopy-referral patient population with digestive symptoms between 2003 and 2012

**Fig. 1:**
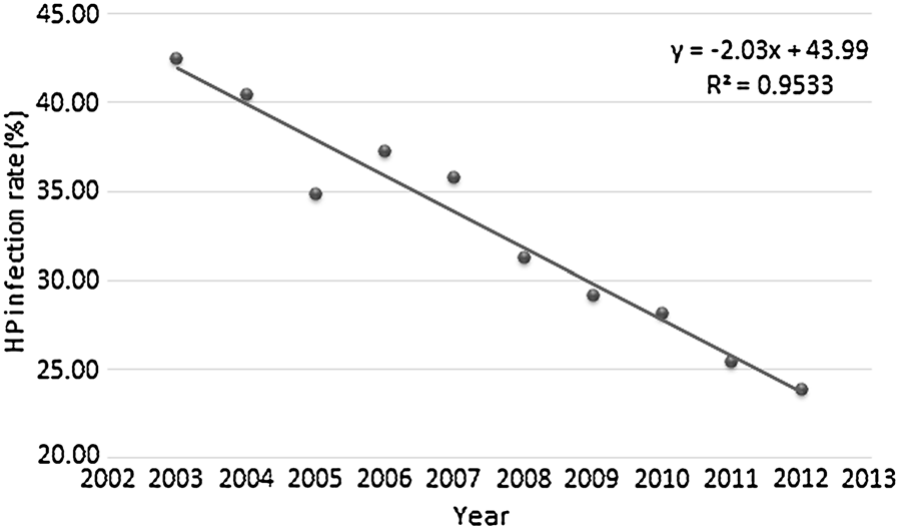
Linear regression analysis of *H. pylori* infection versus time (2003–2012). The calculated regression equation for the correlation of *H. pylori* infection (%) versus time in years from 2003 to 2012 was y = − 2.03x + 43.99

A total of 134,812 patients with digestive symptoms who underwent esophago-gastro-duodenoscopy and RUT in the Endoscopy Center of the First Affiliated Hospital of Nanjing Medical University from 2003 to 2012 were recruited into this study. Mean age was 47.49 ± 14.47 years with an age range of 5–100 years, and male to female ratio was nearly 1:1 (67,859 vs. 66,915). In the study population, mean infection rate of *H. pylori* was 31.97 % over a ten-year period, which dramatically declined from 42.40 % in 2003 to the lowest infection rate of 23.82 % in 2012 (Table 1, *P* < 0.001). There was a marked gender difference in *H. pylori* infection, which was more common in males compared with females (35.20 vs. 28.69 %, *P* < 0.001). The prevalence of *H. pylori* infection gradually increased with age, and peaked at around 20–29 years; then, rates progressively decreased with increasing age. The Cochran-Mantel–Haenszel Chi square test reflected a downward trend in the prevalence of *H. pylori* infection per year (*P* < 0.001, Table 1). Linear regression analysis indicated a negative correlation between *H. pylori* prevalence and time in years (R-Square 0.95, slant-range 43.99, slope −2.03, y = −2.03x + 43.99; Fig. 1). According to this equation, the prevalence of *H. pylori* infection was calculated to decrease by 2 % per year from 2003 to 2012.

**Table 1:** Baseline statistics for age, gender and *H. pylori* infections in UGID patients from 2003 to 2012

Characteristics	Hp (+)		Hp (−)		OR	95 % CI	*P* value
	Number	%	Number	%			
Gender^a^							<0.001
Male	23,883	35.20	43,976	64.80			
Female	19,197	28.69	47,718	71.31			
Age							<0.001
≤19	893	30.99	1989	69.01			
20–29	4426	35.50	8040	64.50			
30–39	9094	34.79	17,043	65.21			
40–49	10,863	33.35	21,712	66.05			
50–59	9786	31.04	21,737	68.96			
60–69	5487	27.94	14,151	72.06			
≥70	2546	26.55	7045	73.45			
Year							<0.001
2003	3458	42.40	4698	57.60			
2004	4602	40.43	6781	59.57			
2005	4274	34.82	8001	65.18			
2006	4647	37.22	7837	62.78			
2007	5269	35.77	9460	64.23			
2008	4402	31.20	9709	68.80			
2009	4528	29.10	11,033	70.90			
2010	4485	28.14	11,454	71.86			
2011	3859	25.41	11,326	74.59			
2012	3571	23.82	11,418	76.18			
CG	30,920	27.41	81,885	72.59			
DU	7280	67.80	3457	32.20	5.58	5.34–5.82	<0.001*
GU	3293	55.70	2619	44.30	3.33	3.16–3.51	<0.001*
GC	1497	33.31	2997	66.69	1.07	1.00–1.13	0.049
RE					0.85	0.82–0.88	<0.001
Presence	4407	28.87	10,859	71.13			
Absence	38,688	32.36	80,858	67.64			

Chi square test was used to calculate the statistical differences of *H. pylori* infections, gender and various UGID groups. The Cochran–Mantel–Haenszel Chi square test was used to identify the liner trend between *H. pylori* infections and years

*CG* chronic gastritis, *DU* duodenal ulcer, *GU* gastric ulcer, *GC* gastric cancer, *DGIMs* diseases of gastrointestinal motility, *RE* reflux esophagitis, Hp *H. pylori*

^a^Since some patient information subsets were incomplete, the sum of patients for some data subsets is less than the total population of 134.812

* The DU and GU groups were compared with patients in the simple CG group without DU, GU and other digestive diseases such as gastric cancer. Compound ulcers (only 543 cases) were assigned into the DU or GU group

**Fig. 2:**
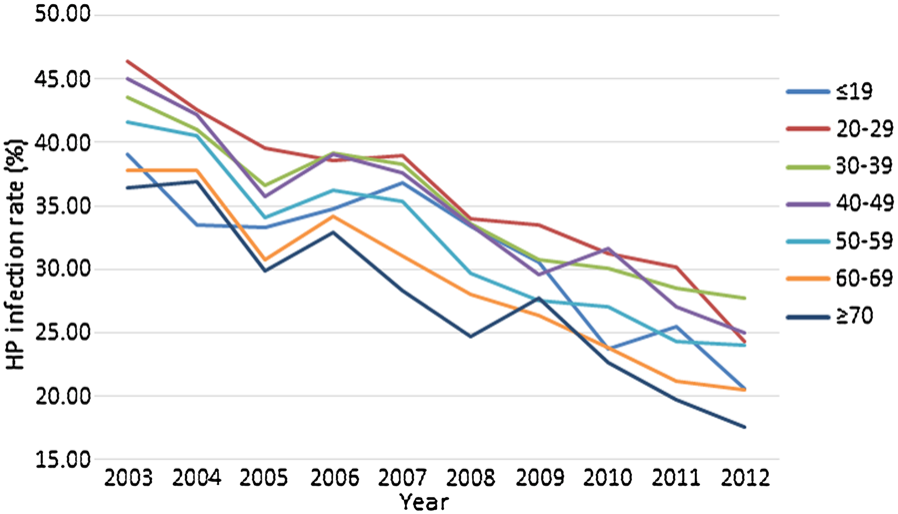
Trend lines for *H. pylori* infection rates in different age groups from 2003 to 2012

In addition to the total *H. pylori* infection rates, the rates of every age group displayed corresponding downward trends in consecutive years between 2003 and 2012 (*P* < 0.001, Cochran–Mantel–Haenszel Chi square test, Table 2). Trend lines of the prevalence of *H. pylori* infection in different age groups were similar among all age groups (Fig. 2), as evidenced by their regression equations calculated by linear regression analysis (data not shown). In summary, the decrease in various age groups mirrored the overall decrease in the *H. pylori* infection rate in a large endoscopy-referral patient population with digestive symptoms.

**Table 2:** *H. pylori* infection rates in different age groups of UGID patients from 2003 to 2012

Age groups	Hp infection rates (%)									
	2003	2004	2005	2006	2007	2008	2009	2010	2011	2012
≤19	39.07	33.47	33.33	34.71	36.77	33.43	30.55	23.73	25.45	20.60
20–29	46.41	42.61	39.57	38.57	38.91	33.97	33.52	31.26	30.14	24.29
30–39	43.55	41.01	36.60	39.19	38.32	33.63	30.78	30.08	28.48	27.68
40–49	45.04	42.13	35.76	39.04	37.61	33.44	29.60	31.63	27.03	25.01
50–59	41.61	40.52	34.07	36.20	35.34	29.73	27.50	27.04	24.34	24.04
60–69	37.78	37.78	30.74	34.13	31.08	28.07	26.37	23.82	21.22	20.53
≥70	36.40	36.90	29.83	32.91	28.33	24.68	27.75	22.69	19.76	17.53

Cochran–Mantel–Haenszel Chi square test was used to evaluate the liner trend of *H. pylori* infection rates in age groups between 2003 and 2012

Hp *H. pylori*

### Changes in the prevalence of UGIDS correlated with *H. pylori* infection rates in UGIDs in an endoscopy-referral patient population with digestive symptoms

**Fig. 3:**
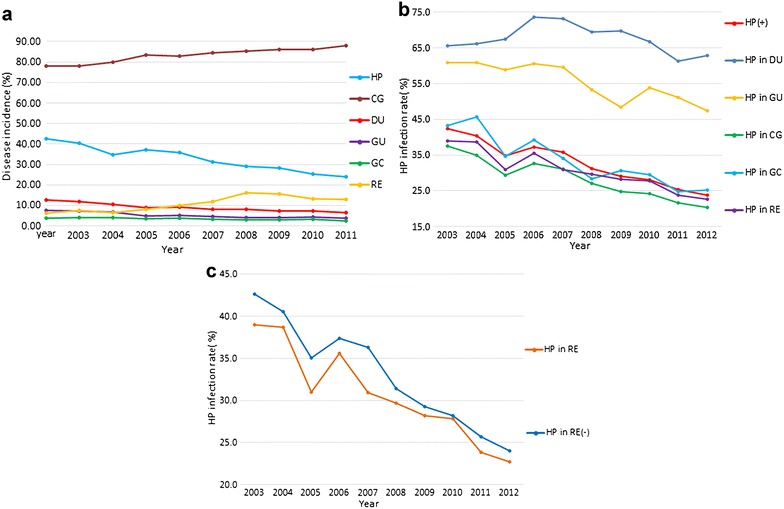
*H. pylori* infection rates and *upper* gastrointestinal diseases (UGIDs) from 2003 to 2012. **a** Disease prevalence (%) of UGIDS (*CG* chronic gastritis, *DU* duodenal ulcer, *GU* gastric ulcer, *GC* gastric cancer) between 2003 and 2012. **b** *H. pylori* infection rates (%) in UGIDs between 2003 and 2012. **c** *H. pylori* infection rates (%) in both positive and negative groups of RE (reflux esophagitis) between 2003 and 2012 are shown

Mean prevalence of simple CG was 83.68 %, which slightly increased from 77.91 to 87.84 % between 2003 and 2012 (Table 3; Fig. 3a). With regard to frequent UGIDs, yearly detection rates of DU and GU also significantly declined within the evaluated ten-year time span from 12.65 to 6.57 % and from 7.51 to 3.78 %, respectively; while the prevalence of GC decreased from 3.76 to 2.34 %. In contrast, RE prevalence rates increased from 6.19 to 12.80 %. Thus, prevalence rates of several UGIDs changed during the ten year evaluation period (Table 3; Fig. 3a).

**Table 3:** *H. pylori* infection rate in UGIDs and incidence of UGIDs between 2003 and 2012

Year	Hp % in UGIDs					UGIDs %				
	CG	DU	GU	GC	RE	CG	DU	GU	GC	RE
2003	37.57	65.65	60.85	43.32	39.01	77.91	12.65	7.51	3.76	6.19
2004	34.94	66.25	60.87	45.63	38.67	77.94	11.94	7.22	4.02	7.52
2005	29.38	67.50	58.90	34.64	31.00	80.00	10.54	6.72	3.95	6.52
2006	32.72	73.63	60.62	39.25	35.57	83.52	8.79	4.73	3.43	8.13
2007	31.05	73.23	59.54	34.04	30.91	82.77	9.16	5.10	3.83	9.99
2008	27.07	69.54	53.26	28.39	29.68	84.48	8.05	4.54	3.34	11.91
2009	24.79	69.81	48.44	30.67	28.18	85.37	8.01	4.16	2.98	16.17
2010	24.23	66.70	53.92	29.55	27.81	86.03	7.19	4.10	3.10	15.61
2011	21.66	61.32	51.12	24.74	23.82	85.98	7.34	4.25	3.11	13.27
2012	20.36	62.85	47.39	25.14	22.68	87.84	6.57	3.78	2.34	12.80
Total	27.41	67.80	55.70	33.31	28.87	83.68	8.69	4.98	3.33	11.32

The changes of spectrum of UGIDs and *H. pylori* infection status in these diseases between 2003 and 2012

Hp *H. pylori, CG* chronic gastritis, *DU* duodenal ulcer, *GU* gastric ulcer, *GC* gastric cancer, *RE* reflux esophagitis

*Helicobacter pylori* infection rates in the endoscopy-referral population with UGIDs including CG, DU, GU, GC and RE were 27.41, 67.80, 55.70, 33.31, and 28.87 %, respectively (OR, 95 % CI; all *P* < 0.001; Table 1; Fig. 3b). *H. pylori* infection rates in the DU and GU groups were higher compared with the sample CG group, while rates were lower in positive groups of RE compared with negative groups (all *P* < 0.001, Table 1). However, no difference in *H. pylori* infection rate was observed in patients with GC and in the sample CG group (*P* = 0.049, Table 1). Furthermore, *H. pylori* infection rates in groups of the above-mentioned UGIDs presented downward trends in some degree as time passed except DU between 2003 and 2012, which decreased from 65.65 to 62.85 % (Table 3; Fig. 3b). *H. pylori* infection rates in both positive and negative groups of the above-mentioned DGIM (RE) presented downward trends over time (Fig. 3c), which corresponded with the decline in *H. pylori* infection rates between 2003 and 2012. However, interestingly enough, *H. pylori* infection rates in the positive groups were always lower than in the negative groups (Table 4; Fig. 3c).

**Table 4:** Correlation of *H. pylori* infection rate and reflux esophagitis (RE) between 2003 and 2012

Year	RE		
	Hp in RE (+) %	Hp in RE (−) %	*D* value %
2003	39.01	42.62	−3.61
2004	38.67	40.57	−1.90
2005	31.00	35.08	−4.08
2006	35.57	37.37	−1.80
2007	30.91	36.31	−5.40
2008	29.68	31.40	−1.72
2009	28.18	29.28	−1.10
2010	27.81	28.20	−0.39
2011	23.82	25.66	−1.84
2012	22.68	23.99	−1.31
Total	28.87	32.36	−3.49

The *H. pylori* infection rate in positive and negative groups of RE between 2003 and 2012

Hp *H. pylori*

## Discussions

Numerous studies reported the decrease in the prevalence of *H. pylori* infection in the US (McJunkin et al. 2011; Elitsur et al. 2009). However, the prevalence of *H. pylori* infection is relatively low in developed countries, which decreased from an ubiquitous infection to under 10 % in the early twenty-first century (Chen and Blaser 2008; den Hoed et al. 2011). As a developing country, China has shown a high prevalence of *H. pylori* infection of more than 60 % in patients with dyspepsia in the late twentieth century (Li et al. 1991). One study in Guangzhou reported that age-standardized *H. pylori* sero-prevalence rate in healthy persons has significantly decreased from 62.5 % in 1993 to 49.3 % in 2003 (Chen et al. 2007). In this present study, mean prevalence of *H. pylori* infection in the endoscopy-referral patient population in Southeastern China was 31.97 %, and it mirrored a marked significant linear decline trend from 42.40 % in 2003 to 23.82 % in 2012. Regression analysis revealed a similar decrease in prevalence rates in every age group study at a rate of approximately 2 % per year. Thus, overall prevalence of *H. pylori* infection decreased steadily and rapidly over a ten year period. Interestingly, it appears that *H. pylori* prevalence in the Southeastern region (Jiangsu and Anhui provinces) of China has been declining for more than 10 years according to a previous study; reporting an infection rate of 58.3 % in 1995 as determined by RUT (Zhang et al. 1998). Therefore, the decline rate of *H. pylori* infection per year was approximately 2 % from 1995 to 2003, which is the first recording year for our study. This present study revealed a marked gender difference for *H. pylori* infections; wherein, it was higher in male patients compared with female patients. With regard to age distribution, the prevalence of *H. pylori* infections peaked at approximately 20–29 years of age; since developing countries are characterized by a high acquisition of *H. pylori* infection during childhood, and majority of young adults display chronic infections persisting throughout life.

Notably, extrapolation of the calculated 2 % annual decline rate would diminish the prevalence of *H. pylori* infection to a zero level in approximately 12 years in Southeastern China. Several pathogen infections such as measles, diphtheria and smallpox are declining dramatically, and have even almost disappeared. Blaser and Falkow (2009) proposed that substantial alterations in macroecology including global warming, modernization, socio-economic conditions, widespread antibiotic use (Blaser 2005), and change in social behaviors of human beings that affect the transmission of pathogens are the cause for the disappearance of microorganisms including *H. pylori.* (Blaser 2006, 2011, 2012) Furthermore, improvements in hygiene and living conditions, as well as economic growth and massive pathogen eradication therapies in China, have directly caused infection rates to fall. *H. pylori*, as an ancient microorganism carried by humans for approximately 58,000 years (Linz et al. 2007), may disappear in several decades unless a genetic variation exists to accelerate the transmission rate or mode. On the other hand, drug resistance caused by repeated substandard antibiotic treatments caused many eradication therapies to fail, and *H. pylori* infections in asymptomatic subjects would persist without eradication therapy. All these facts would affect the prevalence of *H. pylori* infections; and an opposing hypothesis that *H. pylori* would persist at low infection rates in Southeastern China was suggested, as observed in developed countries.

*Helicobacter pylori* is a pathogen that causes peptic ulcer diseases (PUD), and a notable decrease in the global prevalence of PUD has been reported (Dutta et al. 2012; Xia et al. 2001b). In this present study, the estimated prevalence of DU and GU was reduced almost by half from 12.65 to 6.57 % and from 7.51 to 3.78 %, respectively, during the ten year study period in the endoscopy-referral population. The recent decline in PUD, especially in DU, is slow compared to the total in *H. pylori* infection; which correlates with the key role of *H. pylori* infections for PUD. However, the gradual decrease of *H. pylori* infection rates in PUD patients emphasizes the role of other risk factors for PUD. It is well established that NSAIDs are independent risk factors for PUD (Chen et al. 2010), and the prolific use of antisecretory agents such as histamine-2-receptor blockers and proton pump inhibitors were likely to be related to the decrease of PUD (Groenen et al. 2009; Wu et al. 2009). Since non-NSAID non-*H. pylori* ulcers were present in 17 % of patients with DU; (Xia et al. 2001a) it seems likely that with the decreased prevalence of *H. pylori* infection, the proportion of *H. pylori*-negative PUD would increase even if the use of those drugs mentioned above remains constant (Chen et al. 2010).

As one of the high-incidence areas of GC, there are approximately 400,000 new cases per year in China, accounting for 42 % of all cases worldwide (Lin et al. 2011). It is well established that *H. pylori* infections lead to GC; which develop slowly into atrophic gastritis and subsequently into intestinal metaplasia in a number of infected subjects (Kawaguchi et al. 1996; Kuipers et al. 1995; Sipponen et al. 1991). And one study inferred that an important component of gastric adenocarcinoma was represented by *H. pylori* evoked T cell mediated immune responses that were inappropriate in terms of time of onset, intensity and target (Amedei et al. 2014). However, only 1–3 % of *H. pylori* infected patients eventually progress to GC (Noto and Peek 2012). Although the prevalence of *H. pylori* infections is diminishing notably over time, GC prevalence did not markedly change in this present study. In general, the effect of *H. pylori* infections on GC does not appear to emerge in the short-term, similar to PUD; since it usually progresses slowly over a prolonged period of time. In addition, our results did not show any noticeable difference in *H. pylori* infection rates in patients with GC and sample CG; suggesting that *H. pylori* exerts its oncogenic effects on the gastric mucosa through a complex interaction between bacterial factors, host factors and environmental factors, as previous studies have revealed.

RE belong to diseases of gastrointestinal motility (DGIMs) (Penaqini 2001). In recent decades, morbidity of RE dramatically increased in both Western and Asian populations (El-Serag and Sonnenberg 1998; El-Serag 2007; Lim et al. 2005) Concomitant with decreased *H. pylori* infection rates, the prevalence of RE progressively increased from 6.19 to 12.80 % from 2003 to 2012 in this present study. Moreover, *H. pylori* infection rates in the positive groups were always much lower than in the negative groups. Lee et al. (2013) reported that the prevalence of RE doubled after the eradication of *H. pylori* infection. Another study discovered that decreasing the prevalence of *H. pylori* infection induced an increasing severity of RE (Chourasia et al. 2011; Jonaitis et al. 2011). Xia et al. investigated 917 patients during an eight-year follow-up study using upper endoscopy; and found that while *H. pylori* infection decreased, reflux became more frequent (Xia et al. 2001b). Based on these findings, *H. pylori* infection appears to have a significant inverse correlation with RE. The exact correlation between the pathogenesis of DGIMs RE with *H. pylori* remains unclear, although numerous hypotheses were put forward to explain their underlying mechanisms. On one hand, gastric acidity begins to diminish after decades of carriage of *H. pylori* and the gradual age-related development of atrophic gastritis (Blaser and Atherton 2004), which caused the stomach to produce a lower acid load; and therefore, less damage to the distal esophagus when reflux occurs. On the other hand, a neuroimmunological anti-inflammatory mechanism may be responsible for the protective effect of *H. pylori* on RE (Shahabi et al. 2008). Moreover, the effects on esophageal gastric motility including those mediated by vagal innervation may also influence *H. pylori* effects in the esophagus (Ogilvie et al. 1985). These theories implied that *H. pylori* gastric colonization may protect infected patients against DGIMs by changing the esophageal gastric duodenal motility and internal environment.

This present study revealed that the decline of *H. pylori* infections has changed the spectrum of UGIDs to a large extent. The prevalence of *H. pylori*-associated diseases decreased, while related DGIMs increased. However, one may speculate that through the significant decline or disappearance of *H. pylori* in other related health outcomes such as obesity, asthma, allergic conditions, type-1 diabetes and autism may be affected. All these disorders have origins in childhood, when the effects of losing *H. pylori* may result in large effects on the development of those diseases (Blaser 2012).

This present study provided valuable data based on a large endoscopy-referral patient population with digestive symptoms, revealing solid data on *H. pylori* prevalence and quantified the decrease in infection rate in Southeastern China between 2003 and 2012. More importantly, this study identified changes in a spectrum of *H. pylori*-associated UGIDs, and explored the exact relationship between *H. pylori* infection and DGIMs. Nonetheless, this current study has several limitations. Firstly, subjects enrolled in this study were not selected from the general population, but from an endoscopy-referral patient population. Thus, there was bias against asymptomatic infected subjects, which may underestimate the true prevalence of *H. pylori* infection to some extent. Secondly, this study was conducted in a University tertiary center; and therefore, selection bias may have been introduced. More precisely, because this study was conducted in an Endoscopic unit, the possibility of clinical heterogeneity was minimized.

## Conclusions

In conclusion, the prevalence of *H. pylori* infection demonstrates a significant decreasing trend over a 10-year study period. It may be speculated that *H. pylori* infections could reach an extremely low prevalence in the near future in Southeastern China, similar to trends in Western study populations. Decreased *H. pylori* infection rate was correlated with gradual but cumulative changes in the prevalence of *H. pylori*-associated UGIDs. However, there is no sufficient evidence substantiating causal relationships between *H. pylori* infection and related DGIMs. Therefore, further comprehensive research is needed to understand the long-term impact of *H. pylori* infection on UGIDs.

## Methods

### Ethics statement

The study was reviewed and approved by the Ethics Committee of the First Affiliated Hospital of Nanjing Medical University. The ethics committee specifically approved that no informed consent was required since data was analyzed anonymously.

### Study population

This present retrospective study screened 196,442 patients with digestive symptoms aged 5–100 years. All patients underwent esophago-gastro-duodenoscopy at the First Affiliated Hospital of Nanjing Medical University between 2003 and 2012. Inclusion criteria included cases diagnosed endoscopically with the following: chronic gastritis (CG), duodenal ulcer (DU), gastric ulcer (GU), gastric cancer (GC) and reflux esophagitis (RE). Exclusion criteria included the following: diagnosis of active gastrointestinal hemorrhage, esophageal and gastric varices, esophageal cancer, duodenal neoplasms and pyloric obstruction; cases without definite diagnosis by endoscopy or pathology; and rare diseases such as gastric lymphoma and eosinophilic gastroenteritis. Records of gastroscopic results from January 2003 to December 2012 were retrieved from the Endoscopy Information System (EIS; Angelwin, Beijing, China). When multiple endoscopies were performed on the same patient, only the first report was included in this analysis. Demographic characteristics (age, gender, etc.) and endoscopic findings of the respective patients who underwent esophago-gastro-duodenoscopy at the Endoscopy Center were recorded. This study was reviewed and approved by the Ethics Committee of the First Affiliated Hospital of Nanjing Medical University. The ethics committee specifically approved that no informed consent was required due to the anonymous analysis of data.

### Endoscopy

Endoscopy has been the primary mode of investigation for upper gastrointestinal complaints in the Endoscopy Center of the First Affiliated Hospital of Nanjing Medical University. All patients underwent conventional gastroscopic examinations using a standard forward-viewing video gastroscope (GIF 240/260; Olympus Optical Co., Ltd., Tokyo, Japan). Patients received topical pharyngeal anesthesia or general anesthesia during the operating period. All parts of the upper gastrointestinal tract (esophagus, stomach and duodenum) were carefully examined by experienced senior endoscopists in our Endoscopy Center. Histological examinations were done by expert gastrointestinal pathologists of the Department of Pathology, the First Affiliated Hospital of Nanjing Medical University. All participants who underwent endoscopic examinations provided written informed consent before the procedure.

### Diagnosis of *H. pylori* infection and UGIDs

*Helicobacter pylori* infection status was determined based on RUT (Rapid urease test kit, HPUT-H102; Fujian Sanqiang Biochemical Co. Ltd., Sanming, China) results, which has been previously described with a sensitivity of 99 % and specificity of 100 % as a single test (Wong et al. 2001). One biopsy specimen was taken for RUT from either the greater or lesser curvature of the antrum, which was about 3 cm away from the pylorus. DU, GU and RE were diagnosed by endoscopy. DU and GU were endoscopically defined as having a visible mucosal defect with a diameter of ≥3 mm and a depth ≥0.5 mm (Xia et al. 2001a). Compound ulcers (only 543 cases) were assigned into the DU and GU groups, rather than being singled out. The Montreal definition published in 2006 defines gastroesophageal reflux disease (GERD) as a condition that develops when the reflux of stomach contents causes troublesome symptoms and/or complications (Vakil et al. 2006). RE, as endoscopy findings of GERD, was diagnosed as the presence of mucosal erosions within the esophagus and was further validated according to the Los Angeles classification (Lundell et al. 1999). GC was confirmed by histology (negative or positive, grade of neoplasia/dysplasia, and with/without invasion) according to the Vienna classification (Schlemper et al. 2000), which was limited to gastric adenocarcinoma and gastric signet ring cell carcinoma in this study. Multiple biopsy specimens were taken in any area where endoscopists suspected the possibility of GC. Biopsy specimens were fixed in buffered formalin and embedded in paraffin. Slice sections were stained with haematoxylin and eosin. Considering the fact that CG occurs in most of the Chinese population, simple CG was regarded as negative control for DU and GU in this present study. However, patients with peptic ulcers and concurrent gastritis were classified into the DU or GU groups. According to the criteria of the updated Sydney system, CG was diagnosed as a prominent lymphocyte infiltrate or with neutrophil infiltration in the gastric mucosa regardless of *H. pylori* infection (Sonnenberg et al. 2010).

### Statistical analysis

Statistical analysis of data was performed with the Statistical Analysis System software (SAS 9.2, SAS Institute Inc., Cary, NC, USA). Chi square test was carried out for categorical data, and continuous variables expressed as means and ranges were compared with Student’s *t* test. In addition, linear correlation and regression were used to analyze the prevalence trend of various findings, and the correlation between *H. pylori* infection rate and time. Statistical significance of all tests was drawn at *P* < 0.001 in a two-tailed calculation.

## Abbreviations

*H. pylori*: *Helicobacter pylori*
UGIDs: upper gastrointestinal diseases
CG: chronic gastritis
DU: duodenal ulcer
GU: gastric ulcer
GC: gastric cancer
DGIMs: diseases of gastrointestinal motility
RE: reflux esophagitis
GERD: gastroesophageal reflux disease
EIS: endoscopy information system
OR: odds ratio
